# Anti-*Acinetobacter baumannii* single-chain variable fragments show direct bactericidal activity

**DOI:** 10.22038/IJBMS.2022.64062.14106

**Published:** 2022-09

**Authors:** Eilnaz Basardeh, Somayeh Piri-Gavgani, Behnoush Soltanmohammadi, Mostafa Ghanei, Mir Davood Omrani, Mahdieh Soezi, Mohammad Ali Shokrgozar, Masoumeh Azizi, Abolfazl Fateh, Farzam Vaziri, Seyed Davar Siadat, Zahra Sharifzadeh, Fatemeh Rahimi-Jamnani

**Affiliations:** 1 Department of Mycobacteriology and Pulmonary Research, Pasteur Institute of Iran, Tehran, Iran; 2 Microbiology Research Center, Pasteur Institute of Iran, Tehran, Iran; 3 Chemical Injuries Research Center, Systems Biology and Poisoning Institute, Baqiyatallah University of Medical Sciences, Tehran, Iran; 4 Department of Medical Genetics, School of Medicine, Shahid Beheshti University of Medical Sciences, Tehran, Iran; 5 National Cell Bank of Iran, Pasteur Institute of Iran, Tehran, Iran; 6 Molecular Medicine Department, Biotechnology Research Center, Pasteur Institute of Iran, Tehran, Iran; 7 Department of Immunology, Pasteur Institute of Iran, Tehran, Iran

**Keywords:** Acinetobacter baumannii, Antibacterial agents, Colistin, Monoclonal antibody, Phage display library, Single-chain variable-fragment

## Abstract

**Objective(s):**

The high resistance rate of *Acinetobacter baumannii* and the limited number of available antibiotics have prompted a worldwide effort to develop effective antimicrobial agents. Accordingly, identifying single-chain variable fragment antibodies (scFvs), capable of exerting direct antibacterial activity in an immune system-independent manner, may be making immunocompromised patients more susceptible to *A. baumannii* infections.

**Materials and Methods:**

To isolate bactericidal scFvs targeting *A. baumannii*, we panned a large human scFv phage display library against whole-cell extensively drug-resistant (XDR) *A. baumannii* strains grown as biofilm or cultured with human blood or human peripheral blood mononuclear cells plus plasma. The binding of scFv-phages to *A. baumannii* was assessed by the dot-blot assay. Soluble scFvs, derived from the selected phages, were assessed based on their ability to bind and inhibit the growth of *A. baumannii*.

**Results:**

Five phage clones showed the highest reactivity toward *A. baumannii*. Among five soluble scFvs, derived from positive phage clones, two scFvs, EB211 and EB279, had high expression yields and displayed strong binding to *A. baumannii* compared with the controls. Moreover, XDR *A. baumannii* strains treated with positively-charged scFvs, including EB211, EB279, or a cocktail of EB211 and EB279 (200 µg/ml), displayed lower viability (approximately 50%, 78%, and 40% viability, respectively) compared with PBS-treated bacteria.

**Conclusion:**

These results suggest that combining last-resort antibiotics with bactericidal scFvs could provide promising outcomes in immunocompromised individuals with *A. baumannii* infections.

## Introduction

A high ability to survive in harsh conditions and develop resistance to conventional antibiotics has caused *Acinetobacter baumannii* to be considered a significant health threat globally (1-3). Carbapenem-resistant* A. baumannii* (CRAB) infections are linked with a high incidence of morbidity and mortality, with a death rate of as much as 60% for patients with CRAB pneumonia and bloodstream infections (2). Of note, *A. baumannii* has an exceptional capacity to form a biofilm, resulting in persistent and recalcitrant infections (3-5). Given the scarcity of antibiotics for extensively drug-resistant (XDR) *A. baumannii *and the lack of effective therapeutics for patients with pandrug-resistant *A. baumannii *infection, new antibacterial agents are urgently needed (2, 6).

Bactericidal single-chain variable fragments (scFvs) are new antimicrobial biotherapeutics showing significant growth inhibitory activity against some pathogens *in vitro* and *in vivo *(7-12). These antibody fragments, encompassing a light chain variable domain (VL) and a heavy chain variable domain (VH) of a monoclonal antibody (mAb) connected by a linker, have notable characteristics such as appropriate binding ability, substantial tissue penetration, and antibacterial activity independent of the host’s immune system (8, 13). In this regard, several scFvs against *Staphylococcus aureus *(7, 8)*, Pseudomonas aeruginosa *(9, 10), and relapsing fever *Borrelia *(12) were developed, all of which showed significant antimicrobial activity. An scFv with direct bactericidal activity can impact the growth of bacteria through various mechanisms, including interference with biological activities of the bacterium, induction of apoptosis, catalytic activity (e.g., abzyme), or mimicking the bactericidal action of cationic antimicrobial peptides (7, 8, 10-12). Our previous study found three fully human scFvs against *S. aureus*, designated MEH63, MEH158, and MEH183, which showed growth inhibitory activity in *in* *vitro* inhibition assays and a mouse model of bacteremia (8).

*A. baumannii *virulence factors are differentially expressed in various growth conditions (14, 15). We, therefore, sought to identify scFvs that could directly target XDR *A. baumannii* grown under various conditions. In this regard, a large human scFv phage display library was panned against *A. baumannii* grown as biofilm or cultured with human blood or peripheral blood mononuclear cells (PBMCs) plus plasma. Next, five scFvs, derived from phages that showed a significant binding ability to *A. baumannii,* were expressed in Escherichia coli HB_2151_. Two scFv clones (EB211 and EB279) with high-yield expression and strong binding to *A. baumannii* were selected for the growth inhibition assays against *A. baumannii*. 

## Materials and Methods

***Bacterial strains***

Two XDR *A. baumannii* strains, A.b.56 and A.b.58, isolated from an endotracheal tube and the blood of two different patients with *A. baumannii* infections, respectively, were obtained from the Microbiology Department of the Pasteur Institute of Iran (5). Methicillin-resistant *S. aureus* (MRSA) S.a.48, isolated from the cerebrospinal fluid of a patient with *S. aureus *infection were provided by the Department of Mycobacteriology and Pulmonary Researches of the Pasteur Institute of Iran (8, 16). *A. baumannii* ATCC 19606, *Klebsiella pneumoniae* ATCC 700603, and *P. aeruginosa* ATCC 27853 were from the American Type Culture Collection. All strains were routinely cultured in trypticase soy broth (TSB) (Sigma-Aldrich, Saint Louis, USA) or trypticase soy agar (TSA) (Sigma-Aldrich). 

***Susceptibility testing***

The susceptibility of *A. baumannii* ATCC 19606, A.b.56, A.b.58, and *K. pneumoniae* ATCC 700603 to imipenem (Sigma-Aldrich) was evaluated by the broth microdilution method (17). Moreover, the susceptibility of *A. baumannii* strains and *P. aeruginosa* ATCC 27853 (quality control strain) (18) to colistin sulfate (CS; Sigma-Aldrich) was appraised using the broth microdilution method. All results were interpreted based on the breakpoints defined by the Clinical and Laboratory Standards Institute (CLSI) (17). 

***Isolation of A. baumannii-specific scFv-phages***

A large human scFv phage display library (an M13 phage display library; diversity of 2×10^10^) (8, 19, 20) was enriched against *A. baumannii* A.b.56 and A.b.58 in three individual lines. In the first line, the pool of phages (approximately 10^12^ plaque-forming unit [PFU]/ml), amplified from the scFv library, was incubated with the bacteria developing a biofilm following 72 hr (biofilm panning) as previously described (5, 8). In the second and third lines, the purified phages (approximately 10^12^ PFU/ml) from the scFv library were incubated with the bacteria cultured in whole human blood (blood panning) or PBMCs plus plasma (PBMCs plus plasma panning), respectively (5, 8). Whole human blood was obtained from a healthy adult volunteer (male, 50 years). The panning procedure was carried out for four rounds (8). In the next step, the binding ability of output phages (output_1-4_; eluted from each round of biofilm, blood, and PBMCs plus plasma panning) to *A. baumannii* was assessed by the dot-blot assay (polyclonal assay) (8). In brief, 20 μl of bacterial suspensions of *A. baumannii* ATCC 19606, A.b.56, and A.b.58 (approximately 10^8^ CFU/ml) were spotted on the nitrocellulose (NC) membranes (GE Healthcare, Little Chalfont, UK(. Next, the NC membranes were blocked with 5% non-fat milk in tris-buffered saline (TBS) with 0.05% Tween-20 (TBS-T) and then incubated with output_1_, output_2, _output_3_, output_4_, or helper phages (as a control) (New England Biolabs, Carlow, Canada) for one hour at room temperature (RT). Next, the membranes were washed multiple times with TBS-T, followed by addition of horseradish peroxidase-conjugated mouse anti-M13 antibody (M13-HRP) at a dilution of 1:2000 (Santa Cruz Biotechnology Inc, Heidelberg, Germany). After incubation for one hour at RT, the membranes were washed several times with TBS-T, and the signals were visualized by adding diaminobenzidine (DAB; Sigma-Aldrich) and hydrogen peroxide (H_2_O_2_; Merck, Darmstadt, Germany) as chromogen and substrate, respectively. The spotted bacteria incubated with M13-HRP, followed by DAB/H_2_O_2_ or directly incubated with DAB/H_2_O_2_ served as the controls. 

In the monoclonal assay, E. coli TG1 bacteria were infected with output phages from the fourth round of biofilm and blood panning (output_4_) and output phages from the third round of PBMCs plus plasma panning (output_3_), exhibiting the highest signal intensities compared with other output phages and the controls in the polyclonal assay (8). The infected bacteria were plated on Luria-Bertani (LB) agar (Merck) medium with ampicillin (100 μg/ml). After incubation at 37 ^°^C overnight, the colonies were randomly picked and cultured in a TSB medium containing ampicillin (8). The amplified phages were purified and then assessed based on their binding ability to *A. baumannii* A.b.56 using the dot-blot assay, as earlier mentioned in the polyclonal assay (8). 

***scFv expression***

As a first step in the production of soluble scFvs, *E. coli *non-suppressor strain HB_2151 _was infected with the selected scFv-phages (EB204, EB209, and EB211 obtained from biofilm panning and EB279 and EB281 obtained from blood panning) that showed solid binding to *A. baumannii *in the monoclonal assay (8, 20, 21). The bacterial suspensions were then incubated with 0.1 mM isopropyl β-d-1-thiogalactopyranoside (IPTG; GE Healthcare) at 24 ^°^C overnight (8, 21). To release the scFv fragments from the periplasm of *E*. *coli* HB_2151_, the bacterial pellet resuspended in the lysis buffer was incubated on ice for one hour, followed by centrifugation as previously described (8, 21). The expression level of five scFvs (EB204, EB209, EB211, EB279, and EB281) was evaluated by the Bradford assay and sodium dodecyl sulfate-polyacrylamide gel electrophoresis (SDS-PAGE). For immunoblotting analysis, the proteins were electrophoretically transferred from an SDS-PAGE gel (12%) to the polyvinylidene fluoride (PVDF) membrane (GE Healthcare) using a wet/tank blotting system (Bio-Rad, USA), according to the manufacturer’s instructions. The membrane was blocked, followed by a mouse anti-human scFv fragment polyclonal antibody (mhscFvP) incubated at a dilution of 1:200 (8) for one hour at RT. After washing and incubation with a goat anti-mouse immunoglobulin G (IgG) antibody conjugated with HRP (gmAb) at a dilution of 1:2000 (Santa Cruz) for one hour at RT, the membrane was revealed by DAB/H_2_O_2_.

***Sequence analysis***

An overnight culture of *E. coli *HB_2151_ containing the phagemid encoding the scFv (EB204, EB209, EB211, EB279, and EB281) was used for plasmid extraction with the High Pure Plasmid Isolation Kit (Roche Diagnostics GmbH, Mannheim, Germany), according to the manufacturer’s instructions. Following DNA sequencing with forwarding primer 5’-CTA TGA CCA TGA TTA CGA ATT TCT A-3’, the sequences were analyzed by the Gene Runner program, version 6.0 (Hastings Software, Inc., Hastings, NY, USA) and the IMGT V-QUEST database (http://www.imgt.org/IMGT_vquest/analysis) (8, 22). 

***Evaluation of the binding of the purified scFvs to A. baumannii***

The periplasmic extracts of two clones, EB211 and EB279, with the highest expression levels and unique sequences, were purified using nickel-nitrilotriacetic acid (Ni-NTA) resin (Qiagen, Hilden, Germany) as recommended by the manufacturer’s instructions. The eluted fractions containing the scFv fragments were pooled and dialyzed against phosphate-buffered saline (PBS) (pH 7.4) at 4 ^°^C for 24 hr. The purity of scFv fragments was assessed by SDS-PAGE. The binding ability of the EB211 and EB279 scFvs to *A. baumannii* was investigated by the dot-blot assay as described previously by Soltanmohammadi *et al. *(8). Briefly, 20 μl of bacterial suspensions (*A. baumannii* A.b.56, *K. pneumoniae *ATCC 700603,* P. aeruginosa *ATCC 27853, and MRSA S.a.48) (approximately 10^8^ CFU/ml) were spotted on the NC membranes. After blocking, the membranes incubated with EB211 or EB279 (400 μg/ml) for one hour at RT were washed several times with TBS-T. The membranes were then incubated with an mhscFvP, followed by a gmAb. After several times washing with TBS-T, the color reactions were developed with DAB/H_2_O_2_. The bacteria incubated with EB211, followed by an mhscFvP; EB211, followed by a gmAb; an mhscFvP, followed by a gmAb, or DAB/H_2_O_2 _served as the controls. 

Moreover, the binding potential of EB211 and EB279 to human cells was also investigated by the dot-blot assay as previously described (8). In brief, 20 μl of a suspension of human embryonic lung fibroblast MRC-5 cells (approximately 10^7^ cells/ml) (National Cell Bank of the Pasteur Institute of Iran, Tehran, Iran) was spotted on the NC membranes. Next, the membranes were incubated with EB211 or EB279 (400 μg/ml) at RT for one hour. After washing, the membranes were incubated with an mhscFvP, followed by a gmAb. Then, the membranes were washed and developed with DAB/H_2_O_2._ The cells incubated with EB211, followed by a gmAb; an mhscFvP, followed by a gmAb; or DAB/H_2_O_2 _served as the controls.

***Assessment of the antibacterial activities of EB211 and EB279 against A. baumannii***

The growth inhibitory activity of EB211 and EB279 was primarily determined by the microtiter plate assay as previously described (8-11). In brief, 50 µl of *A. baumannii* ATCC19606, A.b.56, and A.b.58 (OD_600_ ) bacterial suspensions were individually incubated with an equal volume of EB211 or EB279 (200 µg/ml) at 37 ^°^C. The bacterial growth was monitored by measuring the OD_600_ every hour for 10 hr and after 20 hr. The effect of the scFv on the growth curve of bacteria was compared with the effect of CS (1 µg/ml), a denatured EB279 (the EB279 scFv heated for 30 min at 100 ^°^C) (200 µg/ml), or PBS on the growth curves of treated bacteria. Furthermore, the agar plate assay examined the bactericidal activity of EB211 and EB279 (8-11). Briefly, 50 µl of *A. baumannii* ATCC 19606, A.b.56, and A.b.58 (OD_600_ ) bacterial suspensions were incubated with an equal volume of EB211 (200 µg/ml), EB279 (200 µg/ml), CS (1 µg/ml), or PBS for five hr (the midpoint of the growth curve of PBS-treated bacteria) at 37 ^°^C. Then, the mixtures were 10-fold serially diluted and plated on LB agar (or LB agar supplemented with imipenem for the XDR strains). After 18-20 hr of incubation at 37 ^°^C, the colonies were counted. Besides, 50 µl of *A. baumannii *A.b.56 (OD_600_ ) bacterial suspension was incubated with a cocktail of EB211 and EB279 (at a final concentration of 200 µg/ml) for five hr at 37 ^°^C. Next, the serially diluted mixtures were plated, and the colonies were counted after 18 hr of incubation at 37 ^°^C. The growth inhibitory activity of EB211 and EB279 against *A. baumannii *A.b.56 was also evaluated at concentrations of 25 and 100 µg/ml by the agar plate assay as mentioned above.

***Evaluation of the antibacterial effects of EB211 and EB279 on K. pneumoniae, P. aeruginosa, and MRSA***

 The bactericidal activity of anti-*A. baumannii* scFvs against *K. pneumoniae*, *P. aeruginosa*, and MRSA was investigated by the agar plate assay (8-11). In brief, 50 µl of *K. pneumoniae *ATCC 700603, *P. aeruginosa *ATCC 27853, and MRSA S.a.48 bacterial suspensions were individually incubated with an equal volume of scFv (EB211 or EB279) (200 µg/ml) for five hours (based on the midpoint of the growth curve of PBS-treated bacteria) at 37 ^°^C. Then, the mixtures were diluted and plated on LB agar (or LB agar containing oxacillin for MRSA S.a.48). The colonies, grown after 18 hr of incubation at 37 ^°^C, were enumerated. The bacteria incubated with PBS served as the control.

***Examination of the amino acid sequence of EB211 and EB279 as polycation agents***

The isoelectric points (pI) of EB211 and EB279 were determined by the ProtParam tool on the ExPASy bioinformatics website (http://web.expasy.org/protparam/) (23). 

***In vitro assessment of scFv-antibiotic combinations***

The antibacterial activity of anti-*A. baumannii* scFvs combined with CS against XDR *A. baumannii* was examined using the checkerboard technique, as previously described (24). In brief, a range of concentrations of CS (0.0625-64 µg/ml) and EB211 and EB279 (3.125-200 µg/ml) was prepared. Next, the bacterial suspension of *A. baumannii* A.b.56, adjusted to approximately 10^5 ^CFU/ml, was treated with CS, EB211, EB279, CS plus EB211, or CS plus EB279 for 24 hr at 37 ^°^C. The uninoculated media and bacteria incubated with PBS served as the controls. The fractional inhibitory concentration index (FICI) for each combination was calculated using the formula “FICI = FIC_scFv _+ FIC_CS_,” where FIC_scFv_ is the minimum inhibitory concentration (MIC) of the scFv in combination/the MIC of the scFv alone, and FIC_CS_ is the MIC of CS in combination/the MIC of CS alone. The combination of scFv with CS is considered synergistic, if FICI ≤ 0.5; additive, if 0.5 < FICI ≤ 2; indifferent, if 2 < FICI ≤ 4; and antagonistic, if FICI > 4 (8, 24).

***Statistical analysis***

Statistical differences between the experimental groups were analyzed by Student’s *t*-test. GraphPad Prism version 6 software (https://www.graphpad.com/) was used for all analyses, and differences were considered statistically significant at *P*-values of < 0.05. 

## Results

***Antimicrobial susceptibility testing***

The antimicrobial susceptibility of three *A. baumannii *strains (*A. baumannii* ATCC 19606, A.b.56, and A.b.58),* K. pneumoniae* ATCC 700603, and *P. aeruginosa *ATCC 27853 is presented in Table 1. *A. baumannii* A.b.56 and A.b.58 were identified as imipenem-resistant strains (MIC: 32 µg/ml). *A. baumannii* ATCC 19606 and *K. pneumoniae* ATCC 700603 were susceptible to imipenem (MIC: 2 and 0.125 µg/ml, respectively). Furthermore, according to the MIC breakpoints of the CLSI for CS (susceptible if MIC ≤ 2 µg/ml and resistant if MIC ≥ 4 µg/ml), *A. baumannii* ATCC 19606, A.b.56, A.b.58, and *P. aeruginosa *ATCC 27853 were considered as colistin-susceptible strains (MIC: 1 µg/ml) (Table 1).

***Selection of scFv-phages specific to A. baumannii***

**Figure 1:**
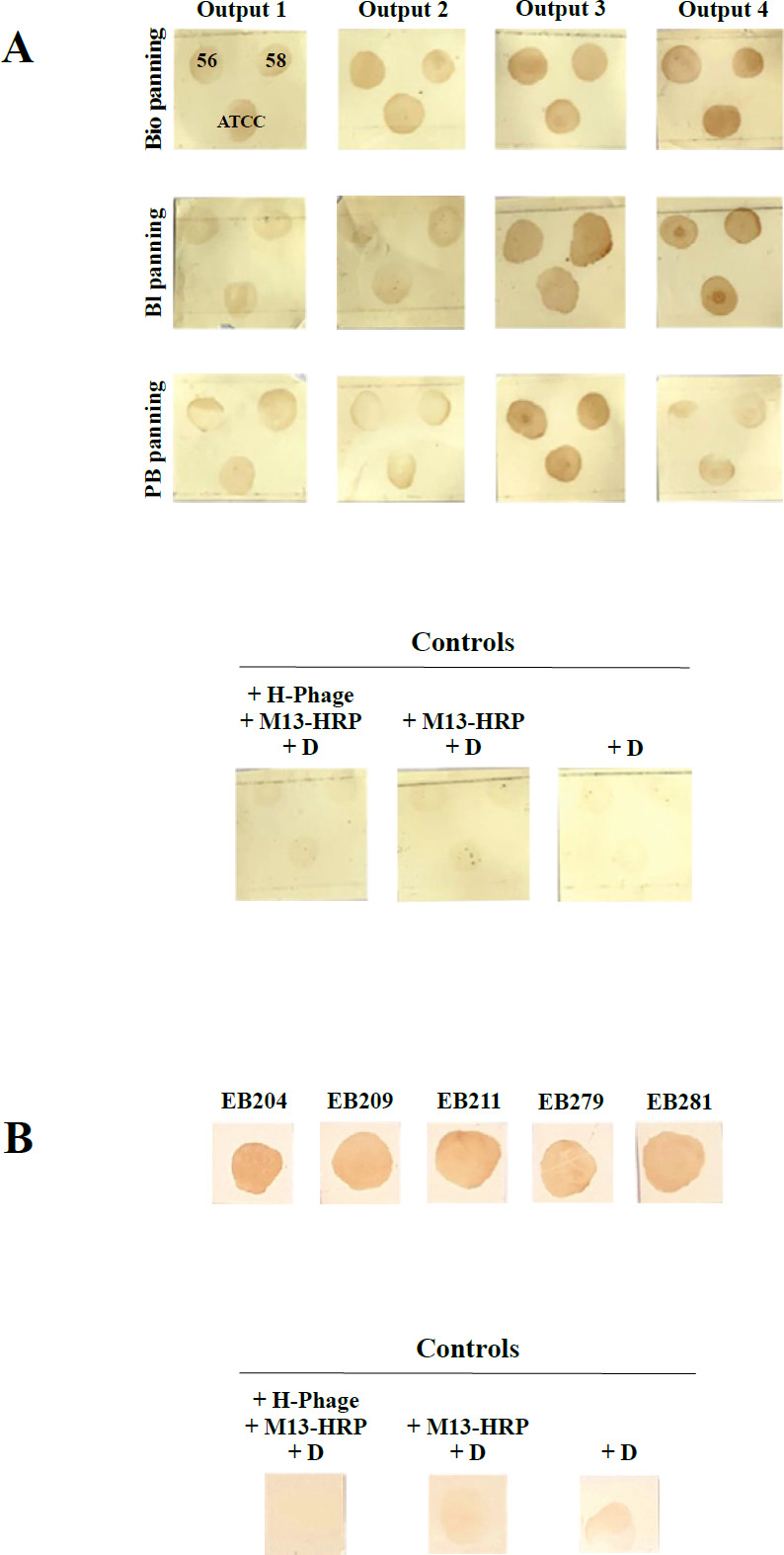
The selected scFv-phages exhibited significant binding to *Acinetobacter baumannii*

A phage-display human scFv library was enriched against *A. baumannii* A.b.56 and A.b.58, grown in different culture conditions. The binding ability of output phages (output_1_-output_4_), obtained from biofilm, blood, and PBMCs plus plasma panning, to *A. baumannii *ATCC 19606, A.b.56, and A.b.58 was evaluated by the dot-blot assay (polyclonal assay) (Figure 1A). The results indicated that output phages from the fourth round of biofilm and blood panning (output_4_) and output phages from the third round of PBMCs plus plasma panning (output_3_) had the highest signal intensities compared with the controls (Figure 1A). 

Moreover, the dot-blot assay was used to determine the binding specificity of scFv-phages from single colonies (220 colonies infected with the output phage obtained from the fourth round of biofilm panning, 200 colonies infected with the output phage obtained from the fourth round of blood panning, and 100 colonies infected with the output phage obtained from the third round of PBMCs plus plasma panning) to *A. baumannii* A.b.56 (monoclonal assay). The results exhibited that the phage clones, EB204, EB209, and EB211, selected from the output phages of the fourth round of biofilm panning, and the phage clones EB279 and EB281, selected from the output phages of the fourth round of blood panning, had significant binding to *A. baumannii *A.b.56 compared with the controls (Figure 1B)*.*

***Binding of the EB211 and EB279 scFvs to Gram-negative bacteria***

**Figure 2:**
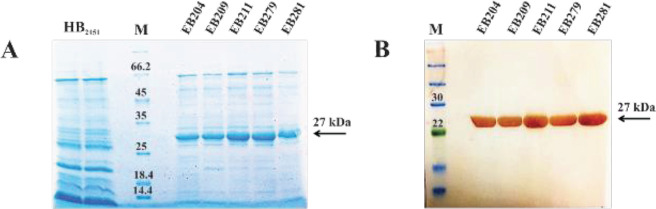
Expression of five soluble scFvs was analyzed by SDS-PAGE and immunoblot assay. (A) SDS-PAGE. Five scFvs (EB204, EB209, EB211, EB279, and EB281) were expressed in *Escherichia coli* HB_2151_ and assessed by SDS-PAGE. Lane M: molecular weight marker. (B) Immunoblotting. The proteins were electrophoretically transferred from a 12% SDS-PAGE gel to the polyvinylidene fluoride (PVDF) membrane, followed by incubation with a mouse anti-human scFv fragment polyclonal antibody. After incubation with a goat anti-mouse immunoglobulin G (IgG) antibody conjugated with horse radish peroxidase, the membrane was developed by DAB/H_2_O_2_. A single protein band corresponding to the scFv was observed at about 27 kDa

The periplasmic extracts of *E. coli *HB_2151_ bacteria, infected with phages amplified from five selected phage clones (EB204, EB209, EB211, EB279, and EB281), were analyzed by SDS-PAGE and immunoblot assay (Figure 2A and B). As illustrated in Figure 2B, a single protein band of about 27 kDa, related to the scFv, was observed in the immunoblot.

**Figure 3:**
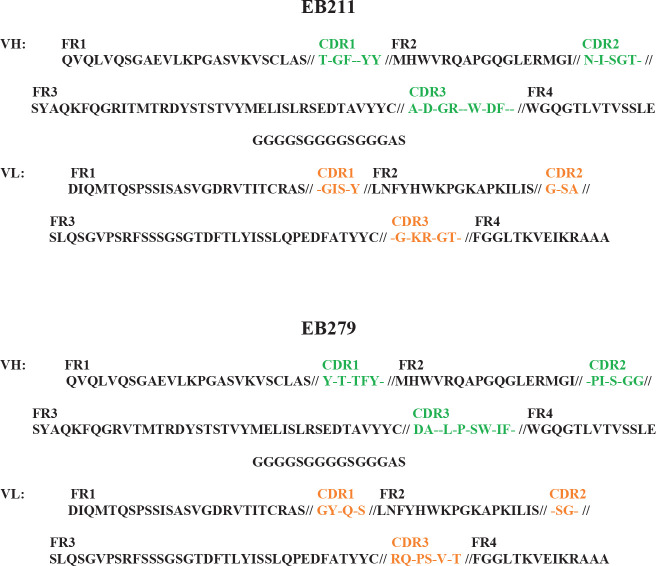
EB211 and EB279 had unique amino acid sequences

Based on the sequencing results, EB204, EB209, and EB211 shared a unique sequence (Figure 3). The sequences of EB279 and EB281 were also similar (Figure 3). Therefore, two scFv clones, EB211 and EB279, which had the highest expression levels, were selected for further examinations. The assessment of the nucleotide sequences of EB211 and EB279 in the IMGT/V-QUEST database revealed that VL and VH of the scFvs belonged to human germline alleles IGKV1-39*01 F and IGHV1-46*01 F, respectively. 

**Figure 4:**
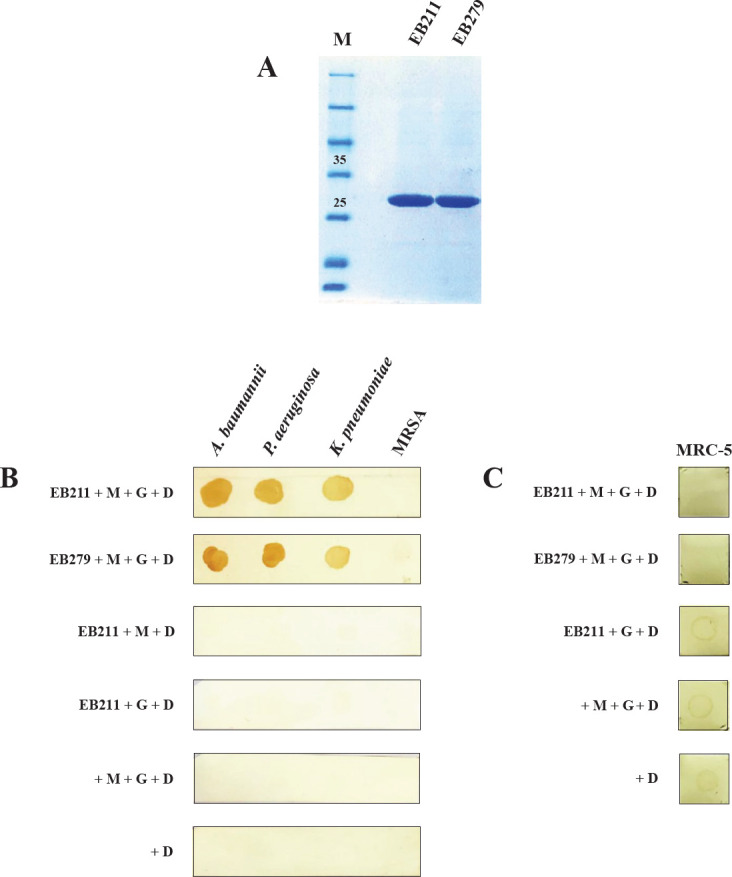
EB211 and EB279 scFvs showed binding to *Acinetobacter baumannii*, *Klebsiella pneumoniae*, and *Pseudomonas aeruginosa*

EB211 and EB279 scFvs were purified using Ni-NTA resin, followed by SDS-PAGE. A single protein band corresponding to the scFv was observed at about 27 kDa (expression yield: 1.8 mg)(Figure 4A). The binding of EB211 and EB279 to *A. baumannii* was investigated by the dot-blot assay. As shown in Figure 4B, both scFvs exhibited significant binding to *A. baumannii* A.b.56, *K. pneumoniae *ATCC 700603, and *P. aeruginosa *ATCC 27853. No binding was observed between the scFvs and MRSA S.a.48, highlighting the binding ability of EB211 and EB279 to Gram-negative bacteria (Figure 4B). Moreover, neither scFvs showed cross-binding to MRC-5 cells (Figure 4C). 

***Bactericidal activity of the EB211 and EB279 scFvs against A. baumannii***

**Figure 5:**
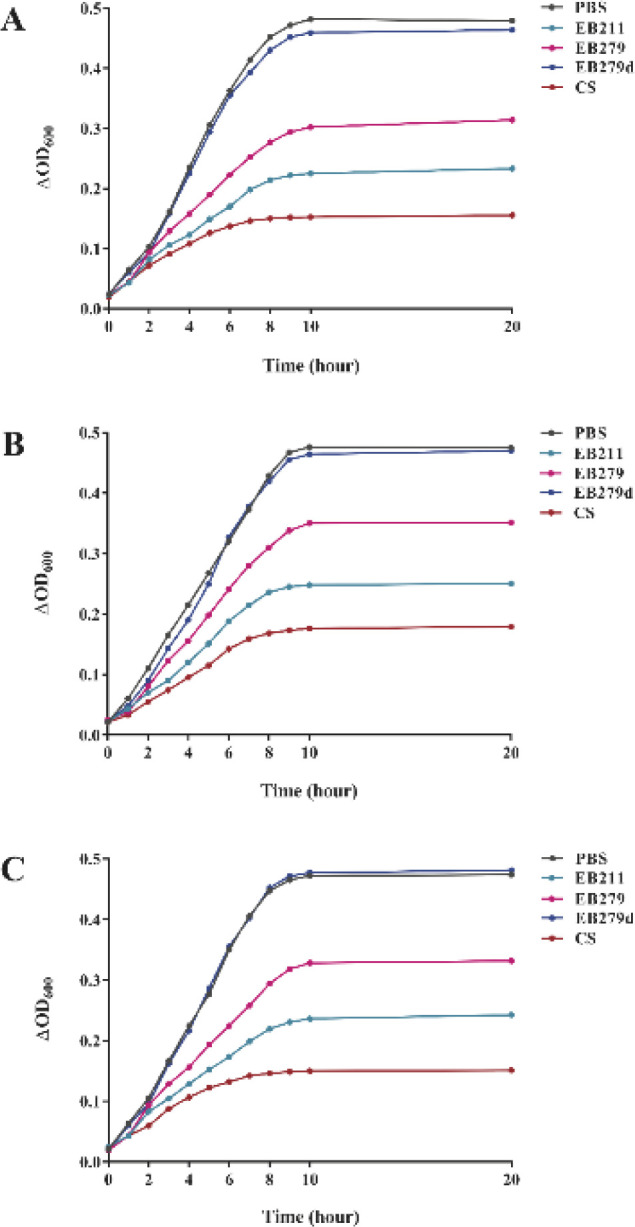
EB211 and EB279 scFvs inhibited the growth of *Acinetobacter baumannii*

 The growth inhibitory activity of EB211 and EB279 was evaluated by the microtiter plate assay. Based on the results, EB211 and EB279 (200 µg/ml) exhibited significant inhibition effects on the growth curves of *A. baumannii* ATCC 19606, A.b.56, and A.b.58 compared with the controls (denatured EB279- and PBS-treated bacteria) (Figure 5A-C). 

**Figure 6:**
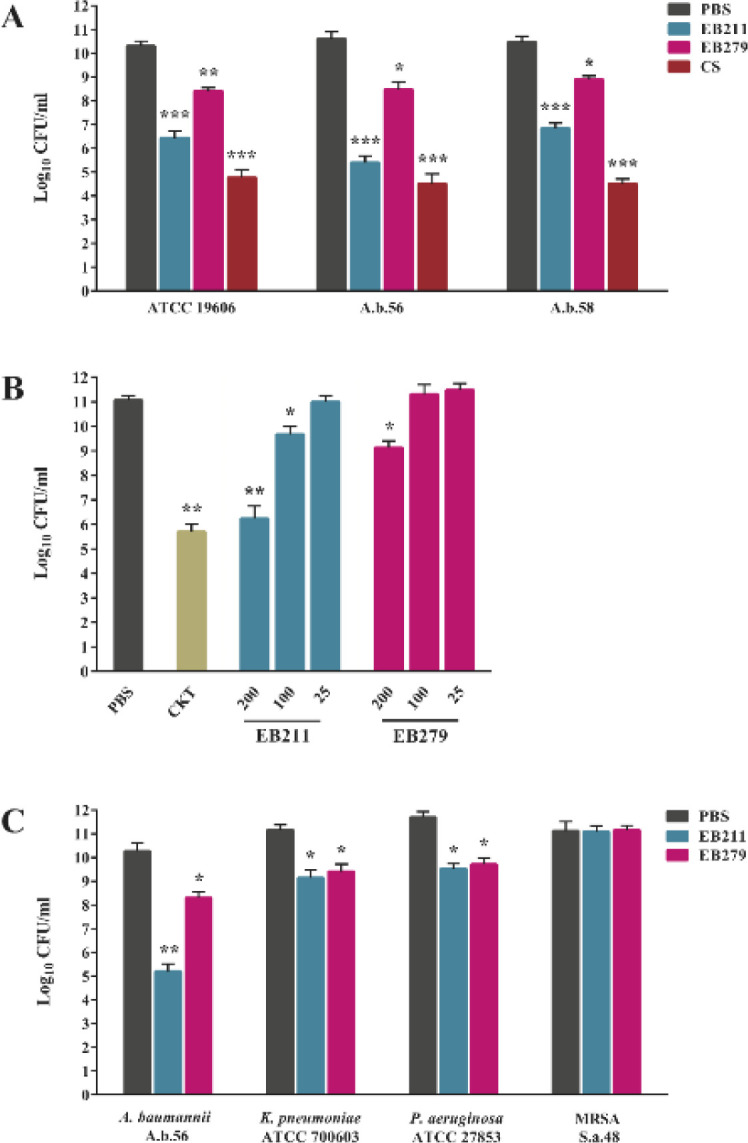
EB211 and EB279 scFvs elicited significant antibacterial activity against *Acinetobacter baumannii*

The agar plate assay also investigated the bactericidal activity of EB211 and EB279 (200 µg/ml) toward *A. baumannii *(Figure 6A-C). As illustrated in Figure 6A, EB211 decreased the viability of *A. baumannii* ATCC 19606, A.b.56, and A.b.58 (approximately 46%, 60%, and 42% reduction, respectively) compared with PBS-treated bacteria. In contrast, EB279 was less efficacious with a mutual effect on the viability of *A. baumannii* ATCC 19606, A.b.56, and A.b.58 (approximately 78% viability) (Figure 6A). Moreover, CS (1 µg/ml) showed the highest bactericidal activity against the three strains tested (Figure 6A). A cocktail of EB211 and EB279 (at a final concentration of 200 µg/ml) exhibited a 60% reduction in the viability of *A. baumannii* A.b.56 compared with PBS-treated bacteria (Figure 6B). The antibacterial activity of EB211 and EB279 toward *A. baumannii* A.b.56 was also appraised at concentrations of 25, 100, and 200 µg/ml. The results revealed that EB211 and EB279 had the highest inhibition activity against *A. baumannii* A.b.56 at 200 µg/ml (Figure 6B). EB211 showed approximately 15% reduction at 100 µg/ml, while no activity against *A. baumannii* A.b.56 was observed at 25 µg/ml. EB279 had no growth inhibitory effect against *A. baumannii* A.b.56 at concentrations of 25 and 100 µg/ml (Figure 6B). As shown in Figure 6C, EB211 and EB279 (200 µg/ml) exhibited a 20% reduction in the viability of *K. pneumoniae *ATCC 700603 and* P. aeruginosa *ATCC 27853 compared with PBS-treated bacteria. On the contrary, no bactericidal effect was observed against MRSA S.a.48 (Figure 6C).

***The potential of EB211 and EB279 as polycation antimicrobials***

 Based on the results from the analysis of the amino acid sequences of EB211 and EB279 in the ProtParam tool on the ExPASys bioinformatic website, both scFvs had a pI of 9.37 and 9.14, respectively, related to the existence of positively charged residues (arginine and lysine; n = 23 and 21, respectively). 

***Combination activity of CS with EB211 and EB279 against A. baumannii***

Based on the data from the checkerboard assay, the combination of CS with EB211 led to a synergistic effect against *A. baumannii *A.b.56, while the CS-EB279 combination displayed an additive effect against *A. baumannii* A.b.56 (Table 2). 

**Table 1:** Minimum inhibitory concentrations (MICs) of imipenem and colistin sulfate against *Acinetobacter baumannii*, *Klebsiella pneumoniae*, and *Pseudomonas aeruginosa *strains

**Strain**	**Antibiotic**	**MIC (µg/ml)**
*A. baumannii *ATCC 19606		2
*A. baumannii *A.b.56	imipenem	32
*A. baumannii *A.b.58		32
*K. pneumoniae *ATCC 700603		0.125
*A. baumannii *ATCC 19606		1
*A. baumannii *A.b.56	colistin sulfate	1
*A. baumannii *A.b.58		1
*P. aeruginosa *ATCC 27853		1

(A) Polyclonal assay. The binding potential of the output phages obtained from biofilm panning (Bio panning), blood panning (Bl panning), and PBMCs plus plasma panning (PB panning) toward *A. baumannii* ATCC 19606, A.b.56, and A.b.58, was evaluated by the dot-blot assay. The bacteria (approximately 10^8^ colony-forming units [CFU]/ml) were incubated with the output phages or helper phages (H-Phage, as a control) (approximately 10^12^ plaque-forming units/ml), followed by a horseradish peroxidase-conjugated mouse anti-M13 antibody (M13-HRP). (B) Monoclonal assay. The binding of the phage clones, EB204, EB209, EB211, EB279, and EB281, to *A. baumannii *A.b.56 (approximately 10^8^ CFU/ml) was shown by the dot-blot assay. All five clones showed intense binding to *A. baumannii* compared with the controls. The bacteria incubated with H-Phage, followed by M13-HRP; M13-HRP; or DAB/H_2_O_2_ served as the controls

Lane M: molecular weight marker

(A) SDS-PAGE. A single protein band corresponding to the purified scFvs was observed at about 27 kDa. Lane M: molecular weight marker. (B) Binding assessment of EB211 and EB279 to *A. baumannii*. In a dot-blot assay, *A. baumannii* A.b.56, *K. pneumoniae* ATCC 700603, *P. aeruginosa* ATCC 27853, and MRSA S.a.48 (approximately 10^8 ^colony-forming units/ml) were individually incubated with EB211 or EB279 (400 µg/ml), followed by a mouse anti-human scFv fragment polyclonal antibody (M) and then by a goat anti-mouse immunoglobulin G (IgG) antibody conjugated with horse radish peroxidase (G). Both scFvs exhibited binding to *A. baumannii* A.b.56, *K. pneumoniae* ATCC 700603, and *P. aeruginosa* ATCC 27853. The bacteria incubated with EB211, followed by M; EB211, followed by G; M, followed by G; or DAB/H2O2 (D) served as the controls.

(C) Binding assessment of EB211 and EB279 to MRC-5 cells. In a dot-blot assay, MRC-5 cells (approximately 10^7^ cells/ml) were incubated with EB211 or EB279 (400 µg/ml), followed by M and then by G. Neither scFvs showed binding to the cells. MRC-5 cells incubated with EB211, followed by G; M, followed by G; or D served as the controls

(A-C) Microtiter plate assay. (A) *A. baumannii* ATCC 19606, (B) A.b.56 and (C) A.b.58 (OD_600_ ≃0.02) were treated with EB211 or EB279 (200 µg/ml) for 20 hr. The growth was monitored by reading the OD_600 _every hour for 10 hr and after 20 hr. The bacteria treated with colistin sulfate (CS) (1 µg/ml), denatured EB279 (EB279d) (200 µg/ml), or phosphate-buffered saline (PBS) served as the controls. The results represent three independent experiments run in triplicate

(A-C) Agar plate assay. (A) *A. baumannii* ATCC 19606, A.b.56, and A.b.58 (OD_600_ ≃0.02) were incubated with EB211 or EB279 (200 µg/ml) for 5 hr. The viability of *A. baumannii* was determined by plating the mixtures on LB agar (or LB agar containing imipenem for the XDR strains) and enumerating colonies after 18 hr of incubation at 37 ^°^C. The bacteria treated with colistin sulfate (CS) (1 µg/ml) or phosphate-buffered saline (PBS) served as the controls. The results represent the mean±SEM of three independent experiments run in triplicate. *P<0.05 and **P=0.01 for EB279 versus PBS; ***P<0.01 for EB211 or CS versus PBS. (B) A cocktail of two scFvs (CKT) (at a final concentration of 200 µg/ml) showed substantial bactericidal activity against *A. baumannii* A.b.56. EB211 displayed weak antibacterial activity against *A. baumannii* A.b.56 at 100 µg/ml. In contrast, EB279 had no activity at concentrations of 25 and 100 µg/ml. The bacteria treated with PBS served as the control. The results represent the mean±SEM of three independent experiments run in triplicate. *P<0.05 for EB211 (100 µg/ml) or EB279 (200 µg/ml) versus PBS; **P<0.01 for CKT or EB211 (200 µg/ml) versus PBS. (C) Both scFvs could affect the growth of *Klebsiella pneumoniae* ATCC 700603 and *Pseudomonas aeruginosa* ATCC 27853. In contrast, they did not affect the growth of MRSA S.a.48. The bacteria treated with PBS served as the control. The results represent the mean±SEM of three independent experiments run in triplicate. *P<0.05 and **P<0.01 for EB211 versus PBS; *P<0.05 for EB279 versus PBS. CFU: colony-forming unit

**Table 2:** Fractional inhibitory concentration index (FICI) values of the combination of colistin sulfate (CS) with EB211 and EB279

**Strain**	**Combination**	**FICI **	**Outcome**
*A. baumannii *A.b.56	CS + EB211	0.5	Synergistic
	CS + EB279	1	Additive

## Discussion

The cell envelope of Gram-negative bacteria (mainly due to the outer membrane) is an advanced and complicated structure, which plays a critical role in the survival and pathogenicity of the bacterium (25-28). In addition to traditional antibiotics, different antimicrobial biologics with various mechanisms of action have been introduced to date, some of which showed promising results, such as bacteriophages and mAbs. Since the emergence of antibiotic-resistant pathogens, phage therapy has gained attention after lying dormant for nearly a century (29). Several studies have reported novel bacteriophages capable of lysing carbapenem-resistant *A. baumannii *(29-31). They demonstrated that these lytic bacteriophages have the potential to be used alone (29, 30) or in combination with colistin (31) in the treatment of *A. baumannii* infections. In contrast, the effectiveness of mAbs in the prevention and treatment of a wide range of disorders and diseases, including autoimmune disorders, cardiovascular diseases, cancers, and infectious diseases, has made mAbs one of the most sought-after biotherapeutics (13). There have been a number of antibacterial mAbs developed against pathogenic bacteria such as *A. baumannii*, *Bacillus anthracis*, *Clostridium difficile*, *S. aureus*, and *P. aeruginosa*, three of which have been marketed (32-38). However, the antibody-dependent enhancement (ADE) of infection, previously reported for viruses such as Ebola virus (39) and severe acute respiratory syndrome coronavirus 2 (SARS-CoV-2) (40), has been observed with the full-length mAbs against *A. baumannii* (36). In contrast, bactericidal scFvs (8, 10-12, 41) with remarkable features, including target-specific binding, small size, excellent tissue penetration, phagocytes- and complement-independent antimicrobial activity (particularly in immunocompromised patients), and subsequently, no ADE effect (Fc-related unwanted events), can be substantial alternatives to conventional mAbs (10, 13, 42, 43). Aiming to isolate bactericidal scFvs specific to *A. baumannii*, a fully human scFv phage display library was panned against live XDR *A. baumannii* grown in various conditions, similar to the growth condition of *A. baumannii* in the human body (whole human blood or human PBMCs plus plasma) or as a biofilm. Accordingly, two scFvs, EB211 and EB279, having unique sequences and showing a significant binding ability to *A. baumannii, *were identified. EB211 and EB279 showed great bactericidal activity against *A. baumannii in vitro* inhibition assays. Among various mechanisms suggested for bactericidal antibodies (e.g., binding to vital proteins, induction of apoptosis, and functioning as AMPs or abzymes) (8, 10-12, 41), compromising the integrity of the cell envelope and disturbing the biological activity of the bacterium are two of the most significant mechanisms reported for bactericidal scFvs in different studies (8, 9, 41). Therefore, we theorized that EB211 and EB279, with net positive charges due to basic residues, likely exerted their bactericidal effect on *A. baumannii* by displacing Mg^2+^ from the lipopolysaccharides and perturbation in the outer membrane (44-46). 

EB211 and EB279 showed moderate binding and growth inhibitory activity against* K. pneumoniae* ATCC 700603 and *P. aeruginosa *ATCC 27853*. *In addition to* A. baumannii*,* K. pneumoniae *and *P. aeruginosa *are the other two members of ESKAPE pathogens (*Enterococcus faecium*,* S. aureus*,* K. pneumoniae*,* A. baumannii*,* P. aeruginosa*, and *Enterobacter species*), which together are the leading causes of many nosocomial infections in the world (47). Hospital-acquired pneumonia caused by *K. pneumoniae* can lead to 50–100% mortality in septicemic or alcoholic patients (48). In a recent study, Mędrzycka-Dąbrowska *et al*. reported that the prevalence of carbapenem-resistant *K. pneumoniae* infections in patients with COVID-19 was up to 53% (49). The opportunistic pathogen *P. aeruginosa* can cause a group of life-threatening infections, including severe skin and soft tissue infections in burn patients, pneumonia in patients on mechanical ventilators or with cystic fibrosis, and bacteremia in patients with compromised immune systems (50-52). In this study, positively charged EB211 and EB279 inhibited the growth of *K. pneumoniae* and *P. aeruginosa*. Consequently, we speculated that the growth inhibitory effect of EB211 and EB279 on *A. baumannii*, *K. pneumoniae*, and *P. aeruginosa*, but not on MRSA, was due to the structural similarity between the cell envelopes of Gram-negative bacteria. 

The use of bactericidal scFvs concurrently targeting several pathogens, in combination with the low dose of antibiotics, some of which cause severe side effects, might lead to promising results in patients (53). Colistin is one of the best therapeutics used to treat *A. baumannii*,* K. pneumoniae*, and *P. aeruginosa *infections; however*,* its application is confined by unwanted side effects (e.g., nephrotoxicity and neurotoxicity) or the emergence of colistin-resistant strains (54). To this end, we assessed the antibacterial activity of CS in combination with EB211 or EB279 against an XDR *A. baumannii *strain (A.b.56). Based on the results, CS showed synergism with EB211 against *A. baumannii* A.b.56, while the combination of CS with EB279 was additive. In a study, the combination of colistin with nisin (an AMP) showed synergistic activity against some XDR *A. baumannii* and colistin-resistant *P. aeruginosa* strains and additive effects against others (55). Nisin could not pass through the outer membrane, restricting its bactericidal effect against Gram-negative bacteria (56). Colistin and polymyxin B increased the permeability of the outer membrane (57), therefore facilitating the penetration of nisin into the bacterium (55, 56). Synergism is mainly observed when two therapeutics have an identical antibacterial mechanism or target site on the bacterium (58). In this study, colistin increased the permeation of EB211 and EB279 into the bacterium based on a self-promoted uptake pathway (57), leading to boosted antibacterial activity of the scFvs, and vice versa. Nevertheless, the combination of EB279 and CS, at ¼ MIC of each agent (59, 60), was not able to elicit a synergistic effect on XDR *A. baumannii *A.b.56, which might arise from the weaker bactericidal activity of EB279 compared with EB211 (based on the results of the growth inhibition assays). 

A cocktail of EB211 and EB279 showed a significant growth inhibitory activity against *A. baumannii*. In the study by Wang *et al*., eight *S. aureus*-specific scFvs were identified, two of which (ZW12 and ZW88) showed the highest growth inhibitory activity against *S. aureus* ATCC 25923 *in vitro *(7). Notably, the combination of eight scFvs had higher antibacterial activity against *S. aureus *inoculum of 10^8 ^CFU/ml than ZW12 and ZW88 alone (7). Using two or more antibacterial antibodies simultaneously has two main advantages (8). First, it is possible to prevent adverse effects by using a lower quantity of each scFv in a cocktail. Second, using a cocktail of two scFvs targeting various sites of the bacterium may preclude the emergence of resistant strains; because the concurrent mutation of factors having critical roles in the vitality of bacteria may lead to detrimental events. 

## Conclusion

In summary, we screened a human scFv phage library on live XDR *A. baumannii *strains, leading to identification of two scFvs, EB211 and EB279. Both positively-charged scFvs showed direct bactericidal activity against *A. baumannii*, which might be due to destabilization of the outer membrane of studied bacteria by displacing Mg^+2^. Furthermore, colistin demonstrated synergistic and additive activity with EB211 and EB279*.* EB211 and EB279 may therefore be promising antibacterial candidates for use in conjunction with colistimethate sodium in treating patients with *A. baumannii *pneumonia*.*

## Authors’ Contributions

FRJ supervised, directed, and managed the study. MG, MDO, MAS, AF, FV, SDS, MA, and ZSH helped design the study. EB, SPG, BSM, and MS performed the experiments. EB, SPG, BSM, and MS were involved in the manuscript preparation. All authors reviewed the manuscript.

## Ethical Approval

Experimental procedures with human blood were approved by the Ethics Committee of the Pasteur Institute of Iran and were done in accordance with the Helsinki Declaration. The participants provided written informed consent before enrollment (Ethics No.: IR.PII.REC.1397.036). 

## Conflicts of Interest

The authors declare no competing interests. 
